# Are the Sick Left Behind at the Peripheries? Health Selection in Migration to Growing Urban Centres in Finland

**DOI:** 10.1007/s10680-020-09568-8

**Published:** 2020-11-04

**Authors:** Maria Vaalavuo, Mikko-Waltteri Sihvola

**Affiliations:** 1 Centre for Health and Social Economics, Finnish Institute for Health and Welfare, Mannerheimintie 166, 00271 Helsinki, Finland; 2 Present Address: Statistics Finland, Helsinki, Finland

**Keywords:** Internal migration, Health care use, Health selection, Mobility, Register data, Urbanisation

## Abstract

We study health selection in rural–urban migration in Finland using register data. Specifically, we ask whether ‘movers’ differ from ‘stayers’ in their use of special health care services prior to moving. We focus on migration to twelve growing urban centres in different sub-groups of the population as well as in different regions, using multinomial logistic regression and multilevel modelling and by distinguishing between short- and long-distance moves. The results show that urban centres attract healthier individuals, while people with health problems are also prone to move, but not to urban centres. The results were similar when looking only at psychiatric diagnoses. The findings suggest that it is important to distinguish between different types of moves when studying health-selective migration. Studying the patterns of migration according to health enables us to understand drivers of regional health differences. Moreover, such evidence will help in projecting future demand for healthcare across the country.

**Electronic supplementary material:**

The online version of this article (10.1007/s10680-020-09568-8) contains supplementary material, which is available to authorized users.

## Introduction

The role of place, residential mobility and migration in health production, health care consumption, and health outcomes are increasingly important in the context of urbanisation. They can affect how health is distributed geographically as well as how services are accessed and used. We already know that there is important variation in the health status of individuals and healthcare expenditure across regions within countries (Smyth 2008; Skinner 2011). Furthermore, evidence shows that migration and demand-side factors are important for understanding these geographical differences (Moura et al. 2019). However, there are also contradictory findings on the associations between health and mobility. Some studies have shown that there is a positive association between poor health and short-distance residential mobility as well as long-distance internal migration (Larson et al. 2004; Tunstall et al. 2014; Green et al. 2015), while others argue the opposite, in that movers are likely to be healthier than those who do not move (Boyle et al. 2002; Andersson and Drefahl 2016; Wilding et al. 2018).

It is often difficult to compare existing studies, as research design and the type of mobility vary from one study to the next. Previous research has typically distinguished between residential mobility and internal migration, where the former refers to short-distance moves (e.g. within neighbourhood and municipality) and the latter to long-distance moves (e.g. migration from rural areas to cities). However, these definitions have often lacked clarity as they have been based on artificially constructed and inconsistent measures such as administrative borders. Our results, based on different types of moves with a rural/urban distinction and length of move as well as different health problems investigated in a single study, bring some clarity to the debate. Our advantage is the use of register data on the total population living in Finland that allows us to overcome many challenges faced by previous studies. Such rich data have not been used in previous studies, so the analysis creates knowledge that is not only relevant for the Finnish audience, but also for an international one.

First of all, previous analyses have often been based on self-reported health, while we use detailed register data on health care use and are able to examine whether the results hold when different diagnoses are looked at. Second, in some studies health is measured *after* moving, even though it is of outmost importance that we can measure it *prior* to moving. Third, survey samples offer limited options for studying associations separately for different population groups. However, it is likely that we will find heterogeneous effects across socio-economic and demographic groups. Finally, robustness analyses have seldom been conducted to investigate the strength of the association in various types of moves. In short, we believe that we are able to contribute to this literature significantly by addressing these limitations. More precisely, we study health selection in rural–urban migration in Finland and ask whether ‘movers’ differ from ‘stayers’ in their use of special health care services prior to moving. We focus on migration to twelve growing urban centres in different sub-groups of the population as well as in different regions, using multinomial logistic regression and multilevel modelling and distinguishing between short- and long-distance moves. We also argue that evidence from diverse settings is necessary to depict the phenomenon more accurately, as previous research on the topic has focused on a limited selection of countries.

Finland offers an interesting case for studying health selection in migration, as the trend in urbanisation in recent years has been particularly strong and the sparsely populated areas in the north and east have experienced a population decline. A handful of cities have witnessed most of the net migration in the country. A special feature of Finland is that it is a geographically large country with long distances between cities. The settlement structure is dominated by one large metropolitan area in the south (Helsinki region) and a number of smaller urban centres also located mostly in the south of the country. According to the OECD functional urban area definition, Finland has in total seven functional urban areas, three of which are classified as metropolitan areas (Helsinki, Tampere, and Turku regions) and four medium-sized areas (Jyväskylä, Kuopio, Lahti, and Oulu regions) (OECD 2018). Compared to other Nordic countries, Finland is noticeably less urban than Sweden and Denmark, although it has been converging to their urbanisation levels since the 1970s (UN Habitat 2018). Finland still remains one of the least urbanised countries in the OECD (2018). The geographical differences in health are also clear (e.g. Martelin et al. 2004), while we do not have evidence regarding to what extent this is driven by selective migration.

The results of this study help us to better understand the drivers of regional health differences in the context of urbanisation. Moreover, such evidence is necessary for projecting future demand for health care across the country. This evidence will also be relevant for decision-makers tackling questions of health inequality, accessibility, and efficient organisation of services. Amid the continuing trend of urbanisation, it will be important to estimate the effects of mobility and migration on the use of services in the areas left behind and in the growing cities.

## Previous Research and Hypotheses

The push and pull theory of moving by Lee (1966) offers a useful framework for analysing factors that may induce an individual to move. According to this theory, individuals’ migration decisions are based on the information they have about their current place of residence in relation to the characteristics of another, a new location, as well as barriers, resources, and personal characteristics that affect one’s mobility. Push factors are the characteristics of the current place of residence that motivate moving away from there, while pull factors are attributes that make the new place a desirable destination for moving.

These push and pull factors are likely to differ depending on the age and life situation of the person. The life course theory of migration seeks to explain a person’s migration decision in relation to different events at different life stages together with preferences, desires, resources, and constraints (e.g. Clark and Lisowski 2017). According to Tyrell and Kraftl (2015), these stages are traditionally conceptualised to include for example infancy, childhood, parenthood, and unmarried and married adulthood, while possible migration events include family migration, student migration, and retirement migration. Individuals and families move to adjust their housing conditions to meet their changing needs as well as resources (Wagner and Mulder 2015; Vaalavuo et al. 2019). For instance, younger people are often found to be more mobile than the rest of the population, and migration rates among young adults are especially high in the Nordic countries (Bernard and Kolk 2019). The higher mobility of younger individuals is often explained by young adults moving to places providing higher education and jobs, as well as families facing new housing needs arising from childbearing or separation (Tunstall et al. 2015; Kulu et al. 2020). Furthermore, a distinction should be made between short-distance *residential mobility* and long-distance *internal migration* that are likely to be affected by different factors and events.

Applying the push and pull theory together with the life course approach of mobility and migration in the rural–urban setting, one can consider growing cities to offer more opportunities in terms of employment, amenities, and services, while congestion, pollution, and a higher cost of living can be seen as push factors. Similarly, rural regions can be seen as inviting relocation destinations due to access to nature and more affordable housing, while limited access to services can hinder one’s propensity to move there. In this vein, the availability of health care services, or the lack thereof, can be regarded as an important factor affecting mobility. Moreover, the extent to which the availability of health care services works as a pull or push factor is likely to depend on the health status and other characteristics of the individual. For healthy and younger individuals, professional career and educational opportunities are likely to be more important factors determining the decision to move to growing cities, while cities offering better access to health care services can also attract individuals with health problems.

Other factors that have been shown to be associated with mobility and migration include employment status and housing tenure: unemployed are more likely to move, whereas owner-occupiers are more immobile than renters (Böheim and Taylor 2002). Maczulskij et al. (2018) found that job displacement increased the probability for intra-regional migration in Finland by 70%, corroborating findings from other European countries. Changes in household size and composition as well as in the household’s economic situation may provoke residential mobility within a shorter range when housing needs and resources change (Rabe and Taylor 2010; Vaalavuo et al. 2019).

Previous research looking at health and residential mobility has looked at (1) the association and causal direction between health and migration and (2) the impact of migration on area-level differences in health, while the two are connected. In this study, we focus on the first one and limit ourselves to looking at how prior health is associated with migration.

Health can be related to residential mobility in a number of ways, and health selection can occur both directly and indirectly: first, healthy individuals of a higher socio-economic status might be more mobile (especially towards big cities) as they move to follow work or educational opportunities; second, the sick might be less likely to move to avoid stress associated with migration; third, individuals with chronic health conditions might be more likely to move closer to health care services; fourth, older people might move closer to health care services (or relatives) in anticipation of future deterioration in health; fifth, lower socio-economic status connected to worse health may affect one’s ability to work and live in a particular location, such as urban centres with higher living expenses; and sixth, health shocks can cause considerable earnings losses and work incapacity (e.g. García-Gómez et al. 2013; Lundborg et al. 2015), which could consequently lead to residential mobility. It is clear that the strong relationship between socio-economic status and health is likely to play an important role in the health/mobility nexus.

Health-selective migration has been studied over a long period of time, but the evidence remains mixed. Some studies have shown that there is a positive association between poor health and residential mobility as well as internal migration (e.g. Larson et al. 2004, for Australian middle-aged women; Tunstall et al. 2014, for the UK): individuals may move in order to be closer to health services or to be closer to their families and the unofficial care they may offer, but poor health may also be connected to other (unobserved) factors that increase the likelihood of residential mobility. To make it more complicated, Green et al. (2015) found that individuals who reported poor health were 20% more likely to move by the following year compared to those in good health, but they were also more likely to move to areas that displayed poorer mortality patterns. Other researchers argue for the ‘healthy migrant’ thesis, meaning that migrants are likely to be healthier than those who do not migrate, and moreover, those who move long distances are healthier than those who move shorter distances (Boyle et al. 2002; Andersson and Drefahl 2016; Wilding et al. 2018).

Furthermore, some studies have shown that psychiatric patients or individuals with mental health problems specifically face greater residential instability than other people with or without health problems (Lix et al. 2006, for Canada; McCarthy et al. 2007, for the US; Tunstall et al. 2014 and 2015, for the UK). There is also an ongoing debate on whether some mental health problems concentrate in urban areas, while the existing rural–urban differences in morbidity are to some extent attributed to socio-economic factors (Paykel et al. 2000; Kovess-Masféty et al. 2005; Peen et al. 2010; Breslau et al. 2014). The difference not explained by observed characteristics of the individuals could be partly explained by selective migration if individuals with psychiatric problems were more likely to move to urban areas. This suggests that different illnesses or disease groups should be looked at separately in a single study. This is why we have added an analysis investigating the connection between psychiatric diagnoses and residential mobility to our study. Psychiatric disorders are a major health issue in the active age population, which also motivates us to look at this specific health problem.

In general, the comparison between existing studies is challenging, as they vary in the measure of ‘short’ and ‘long’ distance, destinations considered, and the health variable used (*inter alia*, self-assessed health, problems with activities of daily living, chronic illnesses, health care visits). Curiously, health selection has not been studied in the context of urbanisation, but usually all types of mobility, including very short within-city migration, any type of residential mobility, or mobility between areas of different deprivation status, have been considered, thus potentially mixing the evidence.

Moreover, the relationship is likely to vary across age and socio-economic groups (Bentham 1988). A number of studies have typically found the propensity to move to be higher among those who are healthier and younger, especially for longer moves (Bentham 1988; Norman et al. 2005; Lu 2008). However, it has also been shown that older individuals with health problems are more likely to move than those with no health problems in the same age group (Bentham 1988; Boyle et al. 2002; Lu 2008; Halliday and Kimmit 2008). There is less evidence of how socio-economic status might modify the association between health and mobility.

Using census data for England and Wales and multilevel logistic regression analysis, Wilding et al. (2018) examined the role of distance in determining health-selective migration. They found that health selection occurs in the range of 20–50 kilometres, but there was no evidence of health selection for distances below 20 kilometres. The authors conclude that further research is needed from other countries to assess the robustness of their findings. Indeed, the definition of ‘long-distance’ is likely to be different in a sparsely populated country with much greater distances between cities, such as in Finland. Also, including the destination type (rural/urban) might affect the results.

Past research dealing with health selection in the Finnish context has been limited. Lankila et al. (2013) used survey data to investigate a cohort of individuals born in Northern Finland and found that dissatisfaction with life and previous health conditions were associated with the likelihood of moving from a rural environment to an urban one. Saarela and Finnäs (2008) analysed internal migration and mortality in Finland using a sample of 40–59-year-old individuals for whom it was possible to identify the place of birth and time of migration. They found some indication of migrants being healthier than non-migrants, but also that the region of birth remained an important determinant of health after migration. Given these mixed results from previous studies and the fact that the authors only had access to a limited scope of data, a more detailed examination of internal migration in Finland is warranted.

The interest in health selection partly lies in its impact on area-level differences in health. For example, in the UK, there is a long tradition of estimating the impact of mobility on geographical differences in health (Boyle et al. 2001; Green et al. 2015; Gartner et al. 2018). Migration may work as a mechanism that drives geographical health inequalities, which have also been observed to be large in Finland (Martelin et al. 2004; THL 2016). This means that it can also be an important driver for diverging health care demands and expenditure.

The results on the impact of migration on health inequalities vary from country to country. In the UK, for example, Norman et al. (2005) found that selective migration is responsible for widening health inequality, as migrants who move away from deprived regions are healthier than stayers. Similarly, Brimblecombe et al. (2000) found that healthier men and women moved away from high mortality areas, while the pattern was more blurred regarding those who initially lived in low mortality areas. Meanwhile, Jongeneel-Grimel et al. (2011) could not identify such an impact in the Netherlands. In the case of Germany, the population left behind was relatively older, less productive, and more prone to developing chronic conditions compared to movers (Westphal 2016).

Findley (1988) has brought up two important points that we will take into account when studying the health–migration relationship: (1) interactive effects and (2) nonlinear association. The first one means that the association between health status and mobility varies across population groups. For example, sick elderly people might be more likely to move, while among younger people residential mobility is more common among the healthy. Nonlinearity, on the other hand, means that people with the best and the worst health could be more likely to move than the others.

## Hypotheses

Based on the previous literature, we test the following hypothesis.

### **Hypothesis 1**

Growing cities attract both healthy younger individuals seeking employment and educational opportunities (especially regarding long-distance moves) as well as individuals with severe health problems seeking better access to health care (especially short-distance moves).

### **Hypothesis 2**

Health selection in urban moves varies across sub-groups of the population.

- **Hypothesis 2a** Among people of prime working age, *better* health is associated with both short- and long-distance moves to urban centres; while among people closer to retirement age, *worse* health is associated with urban moves.
- **Hypothesis 2b** The association between poor health status and migration to urban centres is stronger among higher-income groups with sufficient resources for moving and living in more expensive urban areas.

### **Hypothesis 3**

There is regional variation in the association between health and mobility, as access to health care services as well as employment and educational opportunities vary greatly across the country.

## Data, Methods, and Analytical Strategy

### Data

We use register data of the entire Finnish population from 2014 to 2015.^1^ This data includes information on the exact residential location and postcode of each individual for both years. Based on this information, we can identify moves and calculate the distance between locations in kilometres. Moreover, we have data on the use of public special health care services together with ICD-10 classification of diagnoses based on the patient discharge register and various background characteristics of the individuals and their households (for example age, gender, income, country of birth, labour market status, educational level, housing tenure type).

### Study Population

We limit our study to those aged 18–59 years who resided outside the 12 most populous and growing urban centres in Finland, i.e. in rural areas and small cities with less than 50,000 inhabitants (see Fig. 3 in the “Appendix” for a map). We restrict our analysis to this age group in order to focus on a more mobile group of active age individuals, as older people have been shown to move infrequently (Angelini and Lafferrère 2012). This leaves us with a study population of 1,442,968 individuals, which equals around half the total number of individuals in that age group in Finland.

### Dependent Variable

Our dependent variable is a categorical variable with five possible outcome values based on the type of mobility between 2014 and 2015. We consider mobility by *destination* (rural/urban distinction) and *distance* moved (short/long distinction). First, we believe that the rural/urban distinction plays an important part in mobility decisions, as suggested by Lee’s push and pull theory (1960) as well as theories on life course and migration. Second, we make the distinction between short- and long-distance moves, i.e. residential mobility and internal migration, to analyse whether the findings on the health/mobility connection hold for different distances, as suggested by Wilding et al. (2018).

The first group, which is used as the base category in multinomial logistic regressions, makes up the majority of the study population and includes those individuals who lived outside the urban centres and did not move (‘stayers’). The second and third groups include individuals who moved, but not to locations that were considered urban centres in this study. Such moves were divided into short-distance moves of less than 50 km (‘short rural movers’) and long-distance moves that were longer than that (‘long rural movers’). The fourth group includes individuals who moved a distance of under 50 km to one of the urban centres (‘short urban movers’). Finally, the fifth group includes those who moved a distance greater than 50 km to an urban centre (‘long urban movers’).

### Health Variables

The main explanatory variable in our study is the sum of outpatient special health care visits that a person made during 1 year (2014), but we omitted obstetric-related visits. We do not assume special health care use to be equal to health status, while it is a good proxy for it. To be clear, we use the wording ‘health selection’ and ‘health status’ when we mean selective mobility according to special health care use.

This health variable is categorised into four groups: (1) no visits, (2) 1–3 visits, (3) 4–9 visits, and (4) more than 10 visits during the year. The distribution of health care visits is highly skewed in the population: most individuals in this age group (70.1%) did not have any recorded health care visits during the calendar year 2014, a fifth of the group had 1–3 visits, and very few people had more than 10 visits (3.2%). With the categorisation, we aimed to have reasonably sized groups that allow for testing the nonlinearity hypothesis. We also test for using a continuous variable for health care visits and a binary variable indicating whether the person had used outpatient health care services or not. Robustness analysis was also conducted using a binary variable on the inpatient hospital stays that are likely to refer to more severe health problems. However, only 6% of individuals in the study had inpatients stays in 2014.

We also run the models using psychiatric diagnoses as our health variable to analyse whether different diagnoses lead to different results. Psychiatric diagnoses are a suitable case for a separate analysis as they have been studied before (see literature review above), are among the most common health problems in the active age population, and could affect mobility patterns differently from physical ill-health. We use the ICD-10 coding to distinguish visits with psychiatric diagnoses (all diagnoses in the F-class, that is, all mental and behavioural disorders).

The Finnish health care system is based on a universal tax-funded system in which users pay some out-of-pocket fees. With special health care, we refer to in- and outpatient visits to hospitals. Finnish municipalities have formed 21 hospital districts that provide special health care to their inhabitants.

### Control Variables

All the control variables are measured prior to moving, i.e. in 2014, as the move occurs between 2014 and 2015. The following control variables are used in the study as prior research has shown these factors to be associated with residential mobility in general: age (continuous variable), gender (0 = man, 1 = woman), marital status (0 = not married, 1 = married), housing tenure (0 = not home owner, 1 = home owner), highest educational attainment (1 = lower than upper secondary education, i.e. compulsory education, 2 = upper secondary education, e.g. secondary school graduate, 3 = tertiary education), country of birth (0 = native-born Finn, 1 = not born in Finland), log of disposable equivalised household income, and living in a household with children under the age of 18 (0 = no, 1 = yes). Furthermore, we control for the geographical region (categorical variable of 18 regions) as different regions are expected to display different probabilities for mobility and migration.

An indicator of labour market status was constructed based on information on the main activity of the individual prior to moving. The variable has four categories: (1) individuals outside the labour force (economically inactive individuals, such as retired persons, conscripts, stay-at-home mothers, etc.), (2) employed and self-employed, (3) unemployed, and (4) students.

Our income variable refers to income after taxes and social transfers. It is equivalised using the OECD equivalence scale, which takes into account the size and composition of the household. This scale assigns a value of 1 for the first adult of the household, 0.5 to all other adults in the household, and 0.3 to all children (below the age 14) in the household.

### Methods Used

We use multinomial logit and multilevel modelling to study the association between special health care use and migration and the possible variance across sub-regions. All analyses were conducted using Stata version 15. We use multinomial specification because we want to distinguish between short- and long-distance movers, as well as those who move to growing urban centres as opposed to moving within rural areas. This allows us to compare the determinants of various types of moves. We estimate a set of coefficients corresponding to five outcomes (variable *move*) and we measure change relative to the *k *= 1 group (base category, i.e. not moving) as written in:

$$\ln \left( {\frac{P(move = k)}{P(move = no\,move)}} \right) = B_{k0} + B_{k1} H_{i} + B_{kn} X_{n} + e_{i}$$

where *B*_*k*0_ is the constant term for group *k*, *B*_*k*1_ is the coefficient for group *k*, *H*_*i*_ indicates observed health for individual *i*, *B*_*kn*_*X*_*n*_ is the matrix of covariates for group *k,* and *e*_*i*_ is the error term. We report our results as relative risk ratios and predicted probabilities in different categories of health care visits.

When testing the interactions and in the multilevel model, we simplify our outcome variable by only looking at the odds of moving to an urban centre as opposed to remaining outside of the urban centres in order to make the interpretation of the results easier. We use linear probability models to estimate the interaction effects between age group and outpatient health care visits, as well as income quintile and outpatient health care visits. In the interaction models, we use three age groups (1 = 18–34, 2 = 35–49, 3 = 50–59) and income quintiles based on disposable equivalised income as indicators for age and income instead of the continuous variables used in the main models.

In the multilevel model, individuals are nested in 67 sub-regions of origin that correspond to local labour markets. The model allows us to study whether the outcome and the effect of outpatient health care visits vary from one sub-region to another (i.e. the variation in the random intercept and the random slope respectively). The multilevel modelling aims to disentangle the within-cluster effects from the between-cluster effects (Sommet and Morselli 2017), as we hypothesise that migration patterns are not homogenous across the country.

We compare two models: the first one in which we only estimate the fixed effect of outpatient visits, and the second in which we allow the effect of the health variable to vary across clusters. In both models, fixed intercept and random intercept variance are estimated. In these specifications, we do not simply analyse the chances of moving for individual *i*, but for individual *i* in sub-region *j* as shown in equation below:

$$\log ({\text{odds}})_{ij} = B_{0} + (B_{1} + u_{1j} )*H_{ij} + u_{0j} + B_{n} X_{n} + e_{i}$$

where *B*_0_ corresponds to the fixed intercept, and the random intercept variance for sub-region *j* is indicated by *u*_0*j*_. *H*_*ij*_ is the observed number of health care visits for individual *i* in sub-region *j, B*_1_ is the fixed slope for health visits and *u*_1*j*_ the cluster-specific random slope. *B*_*n*_*X*_*n*_ is the matrix of covariates, and *e*_*i*_ is the error term.

## Results

Table 1 presents background characteristics of the different groups prior to moving (in 2014). We see that movers are younger, especially urban movers, and are less likely to be married or to have children. Short rural movers commonly move only a very short distance (median distance of 2.0 km), while moves to urban centres are clearly longer (more detailed distributions of age, income, and distance moved across categories of movers are presented as histograms in the supplementary material). As for health variables, we see that a larger share of rural movers have used both out- and inpatient health care services compared to stayers or urban movers. In contrast to overall health care use, urban movers more often have a psychiatric diagnosis than those who do not move, while among rural movers the share is even higher.

**Table 1:** Characteristics of the study population (2014) by moving category (2014–2015)

	Stayer	Short rural mover	Long rural mover	Short urban mover	Long urban mover
Mean age (years)	41.1	32.5	30.5	29.5	26.1
Median income (EUR)	25,821	21,670	20,970	25,238	23,019
Median distance moved (km)	0.0	2.0	131.3	22.7	160.2
Woman (%)	48.2	49.7	50.8	50.1	51.8
Employed (%)	78.1	63.1	55.9	63.7	51.3
Student (%)	5.8	12.9	21.7	20.3	31.2
Married (%)	49.4	24.2	19.5	19.0	12.2
Household with children (%)	44.9	39.2	30.1	34.7	30.0
Lowest level of education (%)	16.0	26.7	23.5	23.8	23.1
Home owner (%)	80.0	38.7	45.9	61.0	56.1
Native-born Finn (%)	95.0	92.1	93.2	93.2	92.1
Used outpatient health care (%)	29.1	33.2	31.3	28.5	27.4
Used inpatient health care (%)	5.6	7.0	6.1	4.9	4.5
Psychiatric diagnosis in special health care (%)	2.9	6.3	6.1	4.6	4.6
Number of observations	1,247,657	131,372	18,868	15,712	29,359
Share of the total study population (%)	86.5	9.1	1.3	1.1	2.0

The results, based on multinomial logistic regression analysis in Table 2, suggest that outpatient health care visits are positively associated with mobility within rural areas, but not with moves to urban centres when the number of visits grows. The effect sizes, however, remain small in all cases. Instead, other factors such as labour market status, age, and housing tenure seem to be more important determinants of an individual’s migration decision. Figure 4 in the “Appendix” illustrates predicted probabilities of different types of moves across categories of health care use for an easier interpretation of the results.

**Table 2:** Results from multinomial regression analysis on the association between outpatient health care visits and migration (base category = did not move), relative risk ratios (rrr) shown

	Short rural mover	Long rural mover	Short urban mover	Long urban mover
*Number of special health care visits (ref. no visits)*				
1–3	1.190***	1.135***	1.125***	1.049**
	[1.172,1.209]	[1.093,1.178]	[1.079,1.173]	[1.016,1.083]
4–9	1.233***	1.120***	1.047	0.998
	[1.202,1.264]	[1.055,1.190]	[0.974,1.124]	[0.946,1.054]
10+	1.235***	1.124**	1.084	0.974
	[1.194,1.278]	[1.039,1.217]	[0.985,1.193]	[0.907,1.046]
Age	0.945***	0.939***	0.922***	0.893***
	[0.944,0.945]	[0.937,0.940]	[0.920,0.924]	[0.891,0.894]
Woman	1.194***	1.221***	1.217***	1.307***
	[1.179,1.210]	[1.184,1.259]	[1.176,1.259]	[1.274,1.341]
*Employment status (ref. employed/self*-*employed)*				
Out of labour force	0.907***	1.157***	0.962	1.425***
	[0.886,0.928]	[1.095,1.222]	[0.902,1.027]	[1.363,1.491]
Unemployed	1.033**	1.477***	1.166***	1.322***
	[1.009,1.058]	[1.402,1.556]	[1.090,1.247]	[1.259,1.388]
Student	0.856***	1.602***	1.405***	2.045***
	[0.837,0.876]	[1.529,1.679]	[1.334,1.481]	[1.973,2.120]
Married	0.782***	0.662***	0.572***	0.490***
	[0.768,0.795]	[0.632,0.692]	[0.545,0.601]	[0.470,0.511]
Household with children	1.089***	0.718***	0.769***	0.745***
	[1.073,1.105]	[0.692,0.745]	[0.739,0.799]	[0.724,0.768]
*Education (ref. primary education)*				
Upper secondary education	0.908***	1.137***	1.209***	1.616***
	[0.892,0.923]	[1.091,1.184]	[1.154,1.266]	[1.561,1.673]
Tertiary education	0.800***	1.416***	1.165***	2.243***
	[0.782,0.819]	[1.341,1.495]	[1.094,1.240]	[2.139,2.353]
Home owner	0.215***	0.330***	0.453***	0.491***
	[0.212,0.219]	[0.318,0.342]	[0.435,0.472]	[0.477,0.506]
Foreign born	0.976	1.180***	1.467***	2.000***
	[0.950,1.003]	[1.108,1.258]	[1.368,1.574]	[1.905,2.099]
Log disposable income	1.187***	1.038*	1.643***	1.397***
	[1.169,1.206]	[1.004,1.073]	[1.570,1.718]	[1.354,1.441]
Constant	0.399***	0.136***	0.004***	0.026***
	[0.341,0.466]	[0.098,0.191]	[0.002,0.006]	[0.019,0.036]

Also controlled for the region of origin (18 regions). Number of observations: 1,403,044. 95% confidence intervals in parentheses

***p < 0.001, **p < 0.01, *p < 0.05

Our hypothesis that big urban centres—offering possibly better access to services—would *also* attract individuals with severe health problems is thus refuted for the overall working age population, and it seems that healthy younger individuals following studying or professional opportunities are more likely to make these long moves (*Hypothesis 1*). Interestingly, the correlation between poor health and mobility is the strongest for short rural moves and grows with the number of visits. This could mean that health problems provoke new housing-based needs rather than a need to move closer to services (as the median distance moved in this category is only 2 km). The predicted probability, when keeping other variables at their means, for making a short-distance move within rural areas increases from 5.6 to 6.8% when the number of outpatient health care visits grows from 0 to more than 4. For other groups, the predicted probability remains the same regardless of the number of health care visits.

Interestingly, those with 1–3 visits were more likely to move than those without any visits. This pattern seems to hold even when we restrict our analysis to certain income or age groups (results not shown). However, for other than short rural moves, the difference could be described modest at best. Among short rural movers, it nonetheless shows that even infrequent use of health care services is associated with an increased propensity to move. Considering the literature on the impact of health shocks on earnings and employment, it could mean that changes in economic resources play a role.

We tested alternatively for a continuous and binary variable for health care use (separately for inpatient and outpatient health care use), but this did not change our overall results (Table 5 in the “Appendix”). When using the continuous variable for health care visits, the association was negative between the number of visits and long-distance urban mobility. As for the dummy health variables, the use of health care services was positively associated with mobility, but the effect was smaller for urban moves. The results were similar for both in- and outpatient visits.

For another robustness test (Table 6 in the “Appendix”), we also investigated the association when the definition of urban centre was broadened to include all cities over 50,000 or 38,000 inhabitants. In these cases, the negative association between health care use and long-distance urban migration became slightly weaker. We take this to indicate that it is specifically the biggest growing cities that attract healthy migrants from other parts of the country, and health selection is weaker in smaller cities. (These smaller towns are included in the ‘rural moves’ in our standard analyses.)

The descriptive statistics in Table 1 illustrated that psychiatric diagnoses were more common among both rural and urban movers. When we look at psychiatric diagnoses separately in a multinomial regression model to test whether different diagnoses are differently associated with mobility, we see that outpatient health care visits with a psychiatric diagnosis are positively associated with moves within rural areas and also with moves to urban centres when the number of visits remains under 10 (Table 3). Predicted probabilities show that the probability of a short-distance rural move increases from 5.9 to 8.1% when a person has a small number of outpatient visits with a psychiatric diagnosis, but for other types of mobility there is no visible effect (Fig. 5 in “Appendix”). Thus, the estimates for psychiatric health care use do not seem to differ from our overall estimates shown above.

**Table 3:** Results from multinomial logit regression on the association between psychiatric outpatient visits and migration (base category = did not move), relative risk ratios (rrr) shown

	Short rural mover	Long rural mover	Short urban mover	Long urban mover
*Number of out*-*patient health care visits with a psychiatric diagnosis (ref. no visits)*				
1–3	1.414***	1.420***	1.303***	1.141**
	[1.355,1.476]	[1.294,1.557]	[1.151,1.475]	[1.043,1.248]
4–9	1.382***	1.299***	1.185*	1.189**
	[1.302,1.467]	[1.141,1.479]	[1.004,1.398]	[1.059,1.334]
10+	1.167***	1.199**	1.082	0.951
	[1.106,1.230]	[1.071,1.343]	[0.945,1.238]	[0.856,1.056]

Controls are the same as in Table 2. Psychiatric visits refer to health care visits with a diagnosis in class F in ICD-10 classification. 95% confidence intervals in parentheses

***p < 0.001, **p < 0.01, *p < 0.05

In the interaction models and multilevel analysis, we use a dummy indicating whether the person moved to a growing urban centre or not as an outcome variable. Therefore, for the sake of simplicity, here we are only interested in mobility to urban centres as opposed to remaining in rural areas. When our main analysis is run with this binary outcome variable rather than a categorical variable, we see that people with more outpatient health care visits are less likely to move to urban centres.

**Fig. 1:**
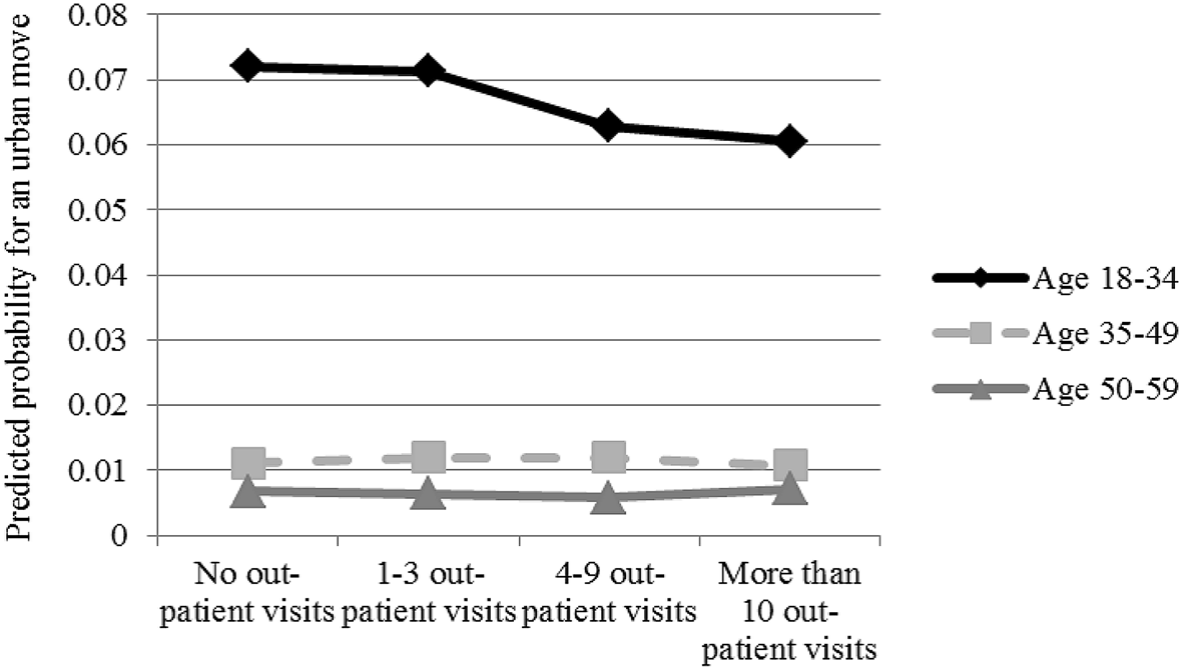
Predicted probabilities for moving to an urban centre, interaction between age and health. *Note*: Predicted probability when other variables are held at their means, based on results from a linear probability model shown in Table 7 in the “Appendix”. Group sizes for movers by age and health care visits are included as supplementary material

One of our aims in this article was to investigate whether health selection in urban moves varies across sub-groups of the population. The results in Fig. 1 show that health selection applies to the youngest individuals only, while among older people the propensity to move rather increases with the use of health services. However, negative health selection among people closer to retirement age is weak at best, as the effect size can be considered rather limited (*Hypothesis 2a*).

**Fig. 2:**
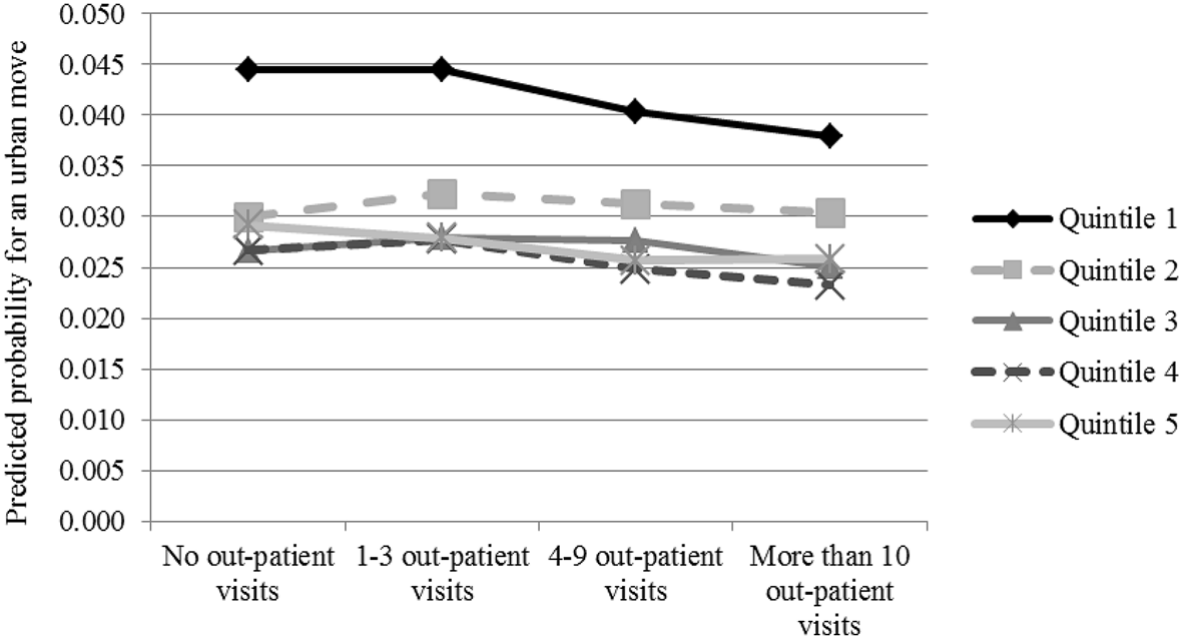
Predicted probabilities for moving to an urban centre, interaction between income and health. *Note*: Predicted probability when other variables are held at their means, based on results from a linear probability model shown in Table 8 in the “Appendix”. Group sizes for movers by income quintile and health care visits are included as supplementary material

**Fig. 3:**
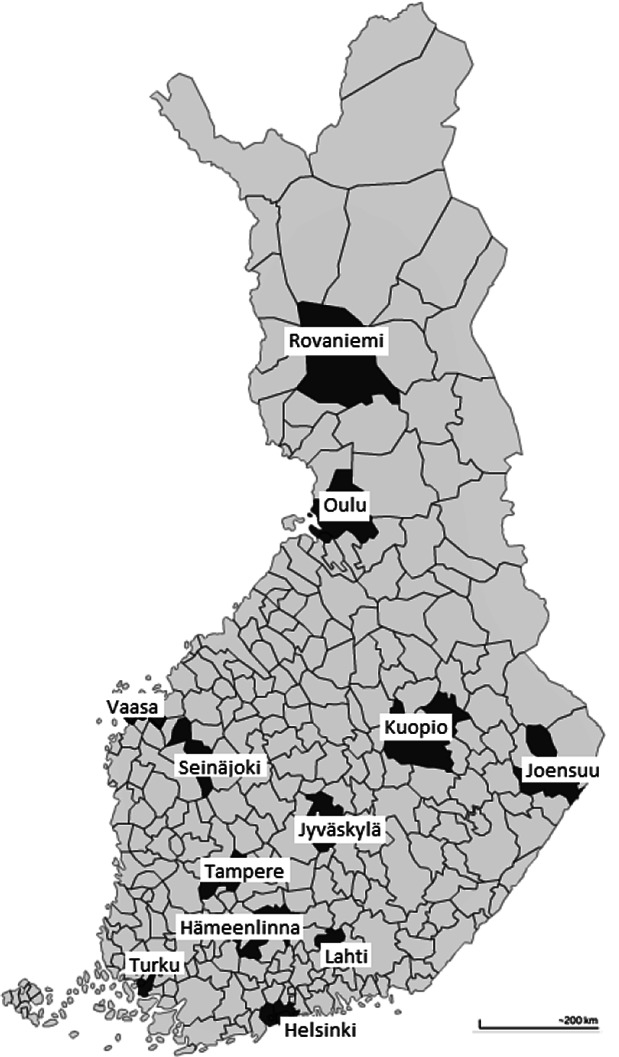
Map of 12 main urban centres of Finland

**Fig. 4:**
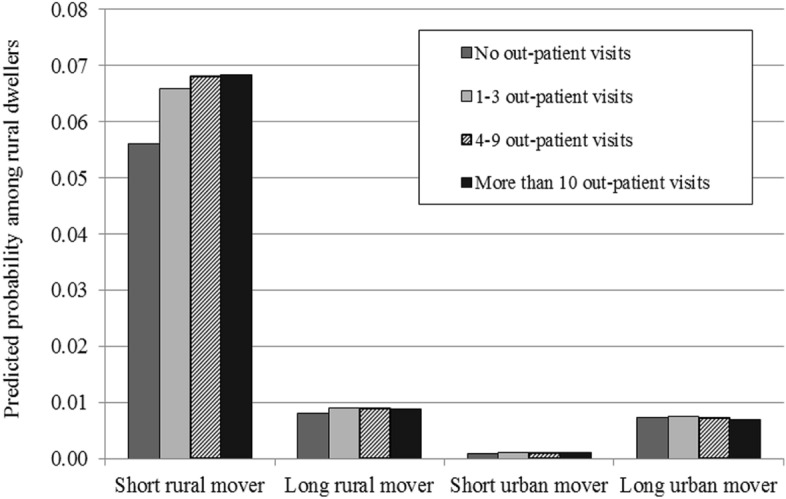
Predicted probabilities for residential mobility by health care use. *Note*: Based on multinomial logit model presented in Table 2

**Fig. 5:**
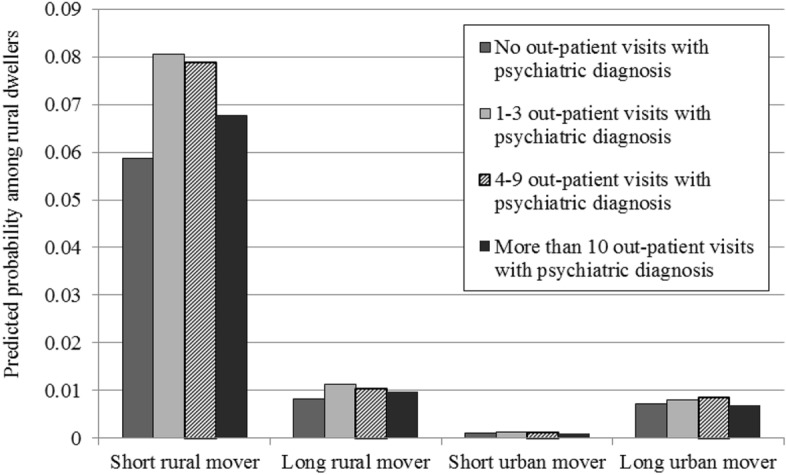
Predicted probabilities for residential mobility by health care use with psychiatric diagnosis. *Note*: Based on multinomial logit model presented in Table 3

Figure 2 presents the results for the interaction between income quintile and health care use. While moving is more common in the bottom income quintile, the pattern of moving according to health care use is rather similar across income quintiles. In all quintiles, the predicted probability for moving decreases with the use of health care services, although the negative association is stronger in the bottom quintile. All in all, there is no support for our *Hypothesis 2b* on the negative health selection among the highest income groups.

In Table 4, we present the results of the multilevel logistic regression. The interclass correlation coefficient indicates that only 2.9% of the chances of moving to an urban centre is explained by differences between sub-regions.^2^ A further analysis suggests that adding a random slope for health care use to the model does not improve the model. (Hence, we do not present the results of the augmented model here.) This means that the effect of health care use is similar across sub-regions and the variance is within one standard deviation of the fixed slope. While the multilevel model indicates that there is random variance in the intercept, i.e. in the probability of moving overall, we reject our *Hypothesis 3* on the variance in the random slope of health care use when other factors are controlled for.

**Table 4:** Results from multilevel logistic regression analysis on the variance across sub-regions in the probability of moving to an urban centre

	Odds ratio	95% confidence intervals
Outpatient health care visits	0.998*	[0.996,1.000]
Age	0.913***	[0.912,0.914]
Woman	1.225***	[1.201,1.250]
*Employment status (ref. outside labour force)*		
Unemployed	0.976	[0.929,1.024]
Employed/self-employed	0.783***	[0.755,0.812]
Student	1.459***	[1.402,1.518]
Married	0.525***	[0.508,0.542]
Household with children	0.741***	[0.724,0.759]
*Education (ref. lower than upper secondary education)*		
Upper secondary education)	1.493***	[1.453,1.534]
Tertiary education	1.813***	[1.747,1.881]
Home owner	0.644***	[0.629,0.660]
Foreign born	1.854***	[1.780,1.931]
Log of disposable income	1.415***	[1.382,1.449]
Constant	0.024***	[0.019,0.031]
Sub-region variance	1.105***	[1.067,1.144]

***p < 0.001, **p < 0.01, *p < 0.05

## Conclusions

In this article, we studied health selection in rural–urban migration in Finland. We distinguished between rural and urban destinations as well as between short- and long-distance mobility. Our main aim was to analyse whether individuals with more outpatient health care visits in 2014 were more or less likely to move from rural areas and small towns to growing urban centres in 2015.

Using rich register data on the total population, this study significantly contributes to the mixed evidence from other countries using survey data, smaller samples, self-reported health, and looking at various types of moves without distinguishing between them. In a single study, we were able to compare different types of moves, analyse nonlinearity and interactions, compare between all health care visits and those with a psychiatric diagnosis, and examine the variance in the effect across sub-regions of the country. Therefore, we believe that our results bring clarity to the contradictory findings presented in the past.

The results show that frequent use of health services is most strongly associated with short-distance (less than 50 km) rural moves, while it reduces the likelihood of long-distance urban migration (more than 50 km). However, the effect sizes are small and socio-economic and demographic factors are the key drivers of migration, and to a large extent they also explain the uncontrolled association between health and residential mobility or internal migration. The results are similar when looking at psychiatric diagnoses.

However, the results suggest that it is important to distinguish between short- and long-distance urban moves as well as moves outside the urban centres. Earlier studies that have shown ill-health to be related to migration have not necessarily made this distinction between different types of moves, which explains the contradictory findings. Indeed, we also find that individuals with more health care visits are prone to move, but not to growing urban centres. Moreover, our robustness check also confirmed that the more restricted the definition of an urban centre, the stronger the finding on health selection. Some deviations from this overall pattern could be observed for different age groups: health selection was the strongest in the youngest age group, while among older people there was a modest increase in the probability to move to an urban centre when the number of health care visits grew. As for income groups, the pattern of health selection was similar across income quintiles.

The study did not find evidence of variance in the impact of health in moving across sub-regions, even though we expected to see differences according to the initial place of residence. In short, our results did not support our hypothesis that people living in remote parts of the country would be more likely to move to urban centres and closer to health care services when facing health problems.

There is some inequality in access to health care across municipalities and socio-economic groups, as individuals with a higher socio-economic status have been shown to use more special health care services when needs are accounted for (van Doorslaer et al. 2006). It is difficult to say how this affects our results as we now saw that poorer people were more likely to move to urban centres and health selection was slightly stronger among them. As a further limitation, the availability of health care services across areas can affect the results but we are not able to account for unmet needs for health care or the use of private services (although limited in special health care in Finland).

Overall, growing urban centres seem to attract healthier and younger individuals. The descriptive findings support the health selection thesis that purports that healthy individuals are more likely to move to follow educational and professional opportunities. This is especially true when we look at longer moves. Therefore, while driven by socio-economic factors, urbanisation can in the short term make the health gap deeper between rural and urban areas, leading to geographical variation in health care expenditure. Previous research from the USA and the Netherlands has already shown that demand-side factors explain a large share of the variation in health care spending and utilisation across regions (Finkelstein et al. 2016; Moura et al. 2019). Studying the patterns of migration according to health enables us to better understand drivers of health differences across regions. Moreover, such evidence will help to project future demand for health care across the country. In the next step, it is important to simulate health care use in places left behind and in the urban centres that attract mostly young and healthy individuals.

Jongeneel-Grimel et al. (2011) argue that health is related to migration mainly through socio-demographic selection, rather than the direct effect of health. We came to a similar conclusion, while the causal impact of health on migration was outside the scope of this study. This is definitely something that should be explored more in future research, while it poses methodological challenges as mobility and migration depend on various factors that can also affect person’s health status as in the case of job loss. However, when we study the association between health and migration, we lose sight of the potential impact migration can have on geographical health differences if we control for all possible socio-demographic and socio-economic characteristics of individuals. Therefore, simple descriptive results should not be overlooked when we want to cast light on the patterns underlying the creation of regional health inequalities.

### Electronic supplementary material

Below is the link to the electronic supplementary material.

Supplementary material 1 (DOCX 92 kb)

## Appendix

See Tables 5, 6, 7 and 8; Figs. 3, 4 and 5.

**Table 5:** Robustness test for different health measures of health care use (base category = did not move)

	Short rural mover	Long rural mover	Short urban mover	Long urban mover
Had used outpatient health care in 2014	1.204***	1.131***	1.104***	1.030*
	[1.188,1.221]	[1.095,1.168]	[1.064,1.146]	[1.002,1.059]
Number of outpatient visits in 2014	1.005***	1.003**	1.003	0.997*
	[1.004,1.006]	[1.001,1.006]	[0.999,1.006]	[0.995,1.000]
Had used inpatient health care in 2014	1.300***	1.229***	1.113**	1.099**
	[1.266,1.335]	[1.154,1.309]	[1.029,1.203]	[1.036,1.166]

Three separate models with different measure of health care use. Controls are the same as in Table 2. 95% confidence intervals in parentheses

****p* < 0.001, ***p* < 0.01, **p* < 0.05

**Table 6:** Robustness test for different definitions of “urban centre” (base category = did not move)

	Short rural mover			Long rural mover			Short urban mover			Long urban mover		
	Urban centre as in our main analyses	Urban centre = all cities above 50,000 inhabitants	Urban centre = all cities above 38,000 inhabitants	Urban centre as in our main analyses	Urban centre = all cities above 50,000 inhabitants	Urban centre = all cities above 38,000 inhabitants	Urban centre as in our main analyses	Urban centre = all cities above 50,000 inhabitants	Urban centre = all cities above 38,000 inhabitants	Urban centre as in our main analyses	Urban centre = all cities above 50,000 inhabitants	Urban centre = all cities above 38,000 inhabitants
*Number of special health care visits (ref. no visits)*												
1–3	1.190***	1.172***	1.168***	1.135***	1.148***	1.182***	1.125***	1.146***	1.159***	1.049**	1.063***	1.065***
	[1.172,1.209]	[1.151,1.193]	[1.145,1.192]	[1.093,1.178]	[1.097,1.201]	[1.123,1.245]	[1.079,1.173]	[1.102,1.193]	[1.113,1.208]	[1.016,1.083]	[1.028,1.099]	[1.028,1.103]
4–9	1.233***	1.234***	1.229***	1.120***	1.156***	1.170***	1.047	1.081*	1.131***	0.998	1.016	1.008
	[1.202,1.264]	[1.200,1.269]	[1.191,1.268]	[1.055,1.190]	[1.076,1.241]	[1.078,1.269]	[0.974,1.124]	[1.011,1.156]	[1.057,1.211]	[0.946,1.054]	[0.960,1.075]	[0.949,1.070]
10+	1.235***	1.237***	1.265***	1.124**	1.155**	1.223***	1.084	1.093	1.140**	0.974	1.009	1.042
	[1.194,1.278]	[1.190,1.285]	[1.211,1.320]	[1.039,1.217]	[1.051,1.269]	[1.099,1.361]	[0.985,1.193]	[0.999,1.197]	[1.039,1.250]	[0.907,1.046]	[0.936,1.086]	[0.963,1.127]

Three separate models with different definition of urban centre. Controls are the same as in Table 2. 95% confidence intervals in parentheses

****p* < 0.001, ***p* < 0.01, **p* < 0.05

**Table 7:** Linear probability model for moving to an urban centre, interaction between age and health

*Number of special health care visits (ref. no visits)*	
1–3	− 0.001
	(0.001)
4–9	− 0.009***
	(0.001)
10+	− 0.012***
	(0.001)
*Age group (ref. 18*–*34)*	
Age group 35–49	− 0.043***
	(0.000)
Age group 50–59	− 0.051***
	(0.000)
*Interaction: health care visits # age group*	
1–3 visits, age group 35–49	0.002
	(0.001)
1–3 visits, age group 50–59	0.000
	(0.001)
4–9 visits, age group 35–49	0.010***
	(0.001)
4–9 visits, age group 50–59	0.008***
	(0.001)
10+ visits, age group 35–49	0.011***
	(0.002)
10+ visits, age group 50–59	0.012***
	(0.002)

Control variables the same as in Table 2. Standard errors in parentheses

****p* < 0.001, ***p* < 0.01, **p* < 0.05

**Table 8:** Linear probability model for moving to an urban centre, interaction between income quintile and health

*Number of special health care visits (ref. no visits)*	
1–3	0.000
	(0.001)
4–9	− 0.004**
	(0.001)
10+	− 0.006***
	(0.002)
*Income quintile (ref. lowest quintile)*	
2. quintile ~ t	0.002**
	(0.001)
3. quintile ~ t	0.005***
	(0.001)
4. quintile ~ t	0.010***
	(0.001)
5. quintile ~ t	0.019***
	(0.001)
*Interaction: health care visits # income quintile*	
1–3 visits, quintile 2	0.002
	(0.001)
1–3 visits, quintile 3	0.001
	(0.001)
1–3 visits, quintile 4	0.001
	(0.001)
1–3 visits, quintile 5	− 0.001
	(0.001)
4–9 visits, quintile 2	0.005**
	(0.002)
4–9 visits, quintile 3	0.005**
	(0.002)
4–9 visits, quintile 4	0.002
	(0.002)
4–9 visits, quintile 5	0.001
	(0.002)
10+ visits, quintile 2	0.007**
	(0.002)
10+ visits, quintile 3	0.005*
	(0.002)
10+ visits, quintile 4	0.003
	(0.003)
10+ visits, quintile 5	0.003
	(0.003)

Control variables the same as in Table 2. Standard errors in parentheses

****p* < 0.001, ***p* < 0.01, **p* < 0.05

## Footnotes

1 Like in other Nordic countries, in Finland all residents have a unique social security or personal number that is used by state agencies for administrative purposes to record various activities, such as hospitalisations, taxation, and social security payments. The data are collected and provided by Statistics Finland, under the licence TK-53-1353-17.

2 Calculation of ICC = var(*u*_0*j*_)/(var(*u*_0j_*)* + *π*^2^/3) [see Sommet and Morselli (2017) for details].
