# Supplementary material for: Are the Sick Left Behind at the Peripheries? Health Selection in Migration to Growing Urban Centres in Finland
Source: Eur J Popul. 2020 Nov 4;37(2):341–66. doi: 10.1007/s10680-020-09568-8 (PMC8035389; doi:10.1007/s10680-020-09568-8)
Supplement: Supplementary file 1 — Supplementary material 1 (DOCX 92 kb) [file 10680_2020_9568_MOESM1_ESM.docx]

**On-line supplementary material**

**Figure S1. Age distribution across moving categories.**


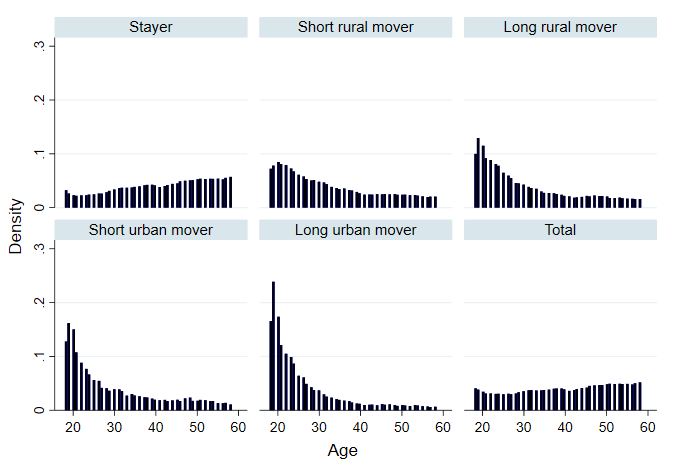


**Figure S2. Income distribution across moving categories.**


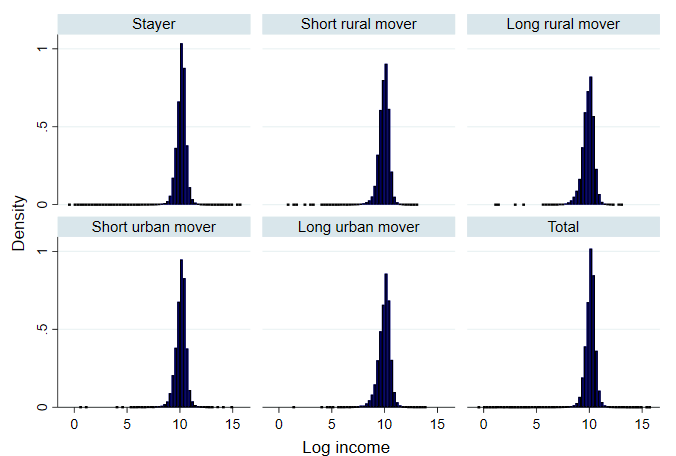


**Figure S3. Distribution of moving distance (in km) across moving categories.**


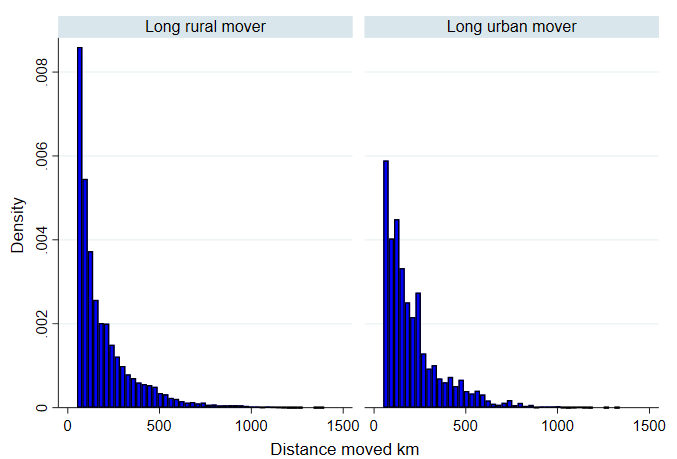


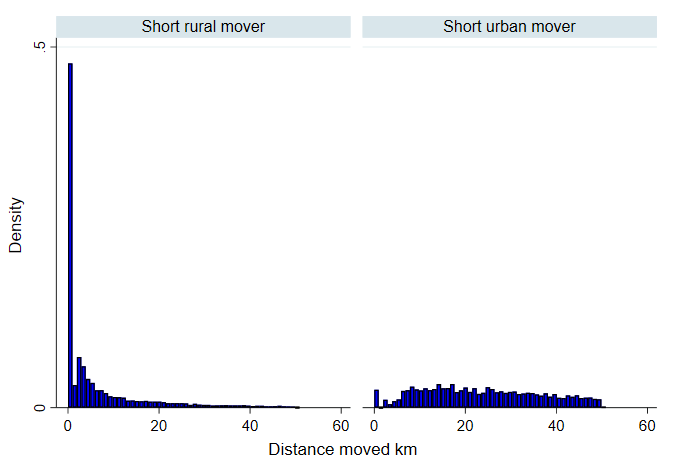


**Table S1. Number of individuals moving to urban centres by age group and income quintile and the number of health care visits.**

|  |  |  |  |  |  |
| --- | --- | --- | --- | --- | --- |
|  | **No out-patient visits** | **1-3 out-patient visits** | **4-9 out-patient visits** | **More than 10 out-patient visits** | **Total** |
| 18-34 | 26,555 | 6,714 | 1,815 | 1,036 | 36,120 |
| 35-49 | 4,280 | 1,281 | 491 | 258 | 6,310 |
| 50-59 | 1,705 | 593 | 216 | 127 | 2,641 |
| *Total* | *32,540* | *8,588* | *2,522* | *1,421* | *45,071* |
| 1 | 7,489 | 2,180 | 696 | 512 | 10,877 |
| 2 | 6,293 | 1,805 | 552 | 333 | 8,983 |
| 3 | 6,055 | 1,585 | 463 | 208 | 8,311 |
| 4 | 5,970 | 1,490 | 372 | 164 | 7,996 |
| 5 | 5,591 | 1,228 | 325 | 149 | 7,293 |
| *Total* | *31,398* | *8,288* | *2,408* | *1,366* | *43,460* |
